# Vicarious ratings of self vs. other-directed social touch in women with and recovered from Anorexia Nervosa

**DOI:** 10.1038/s41598-022-17523-2

**Published:** 2022-08-04

**Authors:** Ashleigh Bellard, Paula Trotter, Francis McGlone, Valentina Cazzato

**Affiliations:** School of Psychology, Faculty of Health, Liverpool John Moores University, Liverpool, UK

**Keywords:** Human behaviour, Neuroscience

## Abstract

Anorexia Nervosa (AN) is an eating pathology characterized by restricted eating, body image distortions and impaired socio-cognitive abilities. Altered responses to affective touch—a pleasant interoceptive stimulus hypothesised to involve activation of the C-Tactile (CT) system, may contribute to the aetiology and maintenance of this disorder. Here, we investigated whether third-party social touch vicarious ratings of different body sites at CT-optimal vs. non-CT optimal velocities differed in women with and recovered from AN (RAN) and healthy controls (HCs). Thirty-five HCs, 27 AN and 29 RAN provided pleasantness ratings for two different tasks designed to probe expectations of how touch is perceived by self (self-directed touch) vs. others (other-directed touch). Findings revealed that both clinical groups, compared to HCs, did not differ in their pleasantness ratings to touch for another but when evaluating touch for self, both clinical groups rated CT-optimal touch as less pleasant than HCs. These findings suggest that AN and RAN women demonstrate an atypical vicarious pleasantness response to affective touch involving self, but not others. Novel therapeutic approaches that help anorexics to better interpret or improve tolerance of affective tactile experiences involving the self may be an important addition to current standard treatments.

## Introduction

Anorexia Nervosa (AN) is an eating disorder typically characterised by body image disturbances, including overestimations in body shape and size^1–3^, atypical eating and dieting behaviours, including maintaining an extremely low and unhealthy Body Mass Index (BMI)^4^. Individuals with AN also show disturbances in interoceptive processing, which is defined as the understanding of internal bodily states such as taste, hunger, stomach distention, pain, heartbeat, and gut attention^5–9^. They also abnormally interpret the internal physiological condition of their body such as sensations of hunger and pain^10^. A mismatch when combining information from internal and external bodily sensations may contribute towards the aetiology of AN^9–11^.

Prior investigations examining the link between interoceptive deficits and AN have mainly concentrated on unrewarding interoceptive signalling, including pain (thermal), neutral interoceptive signalling (heart rate), and interoceptive signals specific to the symptoms of AN (hunger and taste), with only few studies focussing instead on rewarding interoceptive stimuli, such as a gentle, caress-like affective touch^12–15^. Affective touch processing is hypothesised to be facilitated by activation of low-threshold mechanoreceptive C-fibres, known as C-Tactile afferents (CTs)^16–19^. CTs optimally respond to gentle stroking in velocities ranging from 1 to 10 cm/s^20^, which is generally perceived as pleasant and rewarding in neurotypical populations, with ~ 3 cm/s being perceived as most pleasant^16,18,20^. A plethora of investigations have highlighted that the Insula Cortex, specifically the posterior region, is activated when an individual is experiencing affective touch to the hairy skin of the arm^21–23^. The posterior Insula is also thought to be the centre for processing and evaluating the hedonic properties of interoceptive cues, and receives information regarding internal bodily states, thus contributing to interoceptive processing^24,25^. Henceforth, affective touch might be considered a sub-modality of interoception, supported by the Insula Cortex, which conveys affective and affiliative aspects of social touch in agreement with the ‘Social Touch Hypothesis’^26,27^.

To the best of our knowledge, only four independent studies have provided so far evidence for an impairment in the affective touch system in individuals with AN (^12–15^, but also see Carey et al.^28,29^ and Cazzato et al.^30^ for evidence on subthreshold Eds). A seminal study by Crucianelli et al.^14^, reported that AN patients (restrictive-type) perceived gentle skin stroking touch at CT-optimal velocities as less pleasant relative to healthy controls, while there was no such difference for CT-suboptimal velocities, thus suggesting that individuals with AN have abnormalities in the perception of the pleasantness of CT-mediated, affective touch. These results were corroborated by a (brain imaging) study by Davidovic et al.^13^ which showed that at behavioural level, AN rated CT-optimal touch as less pleasant than a group of matched HCs. On the other hand, neuroimaging results were controversial. No differences in the activation of the Insula were observed when comparing conditions of gentle touch and no tactile stimulation amongst the two groups of AN and HCs. A further brain imaging study by Bischoff-Grethe and colleagues^12^ assessed brain correlates of anticipation and perception of gentle touch on the forearm and the palm and specifically in individuals who have recovered from AN. The findings of this study show that RAN did not rate the pleasantness of CT-touch as less pleasant than controls and that there were not any CT-specific neuroimaging findings at the group level, thus not confirming the findings of the previous two studies. Finally, a recent study by Crucianelli et al.^15^ showed that clinical groups of AN and RAN anticipated and rated delivered tactile stimuli as less pleasant than HCs. However, the latter difference was not related to the CT optimality of the stimulation. On the contrary, differences in the perception of CT-optimal touch were best explained by individual differences in alexithymia and interoceptive sensibility. All these studies offer an important theoretical advancement in the understanding of affective touch experiences in disordered eating. Yet, the results are inconclusive and leave this research question still fully unanswered. Furthermore, the studies above have only focused on directly experienced touch (with touch delivered to the self mainly on the forearm and palm) thus, neglecting the importance of investigating vicariously experienced touch.

In the general population, it has been reported that viewing others being touched induces vicarious touch experiences, which is based on the recruitment of brain regions, which originally were responsive to first-hand somatosensation, including the somatosensory cortex, the parietal operculum and the insular cortex^31–34^. Furthermore, we have recently demonstrated that ratings of vicarious touch have the same relationship between velocity and anticipated pleasantness as directly felt touch^35^. However, there are several reasons to expect differences in ratings of vicariously experienced touch in the case of AN and particularly when this is self- compared to when this is other-directed. Indeed, interpreting the affective state of two persons exchanging interpersonal touch requires social cognition, such as Theory of Mind (ToM). One important aspect of ToM is cognitive perspective taking which is one’s capacity to compare state of self (1st person perspective) with another individual (3rd person perspective), with both perspectives involving different neural and cognitive processes^36,37^. It is well known that anorexics have difficulties in attributing mental states (intentions, feelings, beliefs) to others and in explaining and predicting others' behaviour, which involves the decoding of affective stimuli^38–44^. Further, fMRI studies have revealed that even after recovery, anorexics have reduced functioning in social and cognitive networks, compared to healthy controls, which again can account for their difficulty in ToM perspective taking^38,45^. Given that previous studies have found reduced pleasantness in individuals with AN during real experience of (self-directed) touch^13–15^, and that observing others receiving touch activates touch experiences and corresponding brain regions^46,47^, it is reasonable to expect that when anorexics are asked to provide third-party ratings of touch pleasantness, they will use their own affective experiences to interpret the feelings generated in another. This could be possibly due to a ‘learned affective experience’ of touch which is guiding their interpretation and inferences of others pleasantness towards touch^48^. For example, if an individual suffering from anorexia observes a sibling receiving touch and positively engaging with that touch, they themselves will be aware that, although they do not like touch, other individuals do. In turn, they will use this learned re-experience to guide their behaviour^48^. Accordingly, one might expect that reduced pleasantness observed during real tactile experience should also extend to vicarious responses to social touch, a hypothesis which has not been investigated yet, therefore leaving an important gap in the literature of vicarious touch experience in AN*.*

## The current study

With the present study we aimed to fill this gap by investigating whether third-party vicarious ratings of social touch delivered at CT-optimal vs. CT non-optimal velocities differ in women reporting current diagnoses of AN. Participants were shown a series of videos with affective touch being delivered to various body sites of actors, after which participants answered two questions: “How much would you like to be touched like that?” (self-directed touch) and “How pleasant do you think that action was for the person being touched?” (other-directed touch). Following our reasoning above, we expected patients with AN to perceive vicarious self-directed tactile stimuli as less pleasant than other-directed touch and compared to healthy controls. Furthermore, we explored whether specific dimensions of body awareness and of social touch experiences, may predict patients’ third-party vicarious pleasantness ratings of touch when delivered to self, compared to when delivered to others^49^. Accordingly, we focused on the ‘Trusting’ subscale of the Multidimensional Assessment of Interoceptive Awareness (MAIA)^50^ given that a previous work by Brown et al.^51^ reported that lower trust in one’s body signals was most robustly associated with ED psychopathology including higher restraint, eating concern, weight and shape concern, and binge eating and purging symptoms. Furthermore, a recent study confirmed that items from the MAIA Trusting subscale were the most central in bridging interoceptive awareness and ED symptoms^52^.

We also focused on ‘Attitude to intimate touch’ of The Touch Experiences and Attitudes Questionnaire (TEAQ)^53^ because previous research reported that AN patients experience intimate stimuli with lower valence and dominance than healthy controls^54^. Furthermore, women suffering from disordered eating report difficulties experiencing closeness with a partner and low levels of satisfaction in relationships^55^, they also express a fear of intimacy^56,57^ and report avoidance of interpersonal relationships with men^58^ due to their lack of ability to form safe and close emotional bonds with others including that of romantic and sexual^57^. Importantly, we also recruited recovered AN (RAN) individuals to examine whether the differences in self- vs. other-directed third-party ratings hypothesized in AN as explained above may also apply to recovered patients, in comparison to HCs. Indeed, by testing women who have recovered from AN, it is possible to draw more solid conclusions about whether any potential abnormality observed in processing tactile pleasure are a cause or a consequence of starvation, thus ruling out the impact of malnutrition in the acute stages of illness which can cause cognitive and emotional deficits per se.

In line with previous evidence demonstrating blunted responses to actual touch experience^12–15^, we hypothesised that self-directed touch would be somewhat less pleasant than other-directed touch. On the contrary, in the case of other-directed touch, we hypothesised that ratings of pleasantness may be in line with controls and RAN women’ responses due to a ‘learned effect’ of the tactile experience as rewarding/pleasurable. Accordingly, it might be plausible that when observing others receiving touch, anorexics might be using a ‘learned’ experience of touch as pleasant for the others to guide their interpretation and inferences of pleasantness^48^. However, given that results of a reduction in the perceived tactile pleasantness in terms of specificity to CT-optimal stroking velocities are to date controversial, we did not make any specific prediction with these regards. Finally, we hypothesized that scores obtained to the MAIA Trusting and to the TEAQ Attitude to intimate touch scales would predict pleasantness ratings for self-directed social touch, for both AN and RAN, but not for HCs.

## Materials and methods

### Participants

A total sample of 91 cisgender female participants, assigned to three groups (Current Anorexics vs. Remitted Anorexics vs. Healthy controls) based on self-reported Anorexia Nervosa Diagnosis (Current AN diagnosis, previous AN diagnosis and no AN diagnosis) were recruited for this online study. The total sample size was calculated based on the results of a power analysis using G*Power 3.0.10^59^, which indicated that a minimum sample of 81 participants was needed to detect a medium effect (f = 0.25) with 95% power, using a Mixed ANOVA with alpha at 0.05 (two tailed).

Only females were used in the current investigation, as females are typically more sensitive to touch discrimination and respond more positively to touch than males^60^, and because the incidence of eating disorders is greater in women than men^61^. Twenty-seven were current anorexics aged 18–47 (M = 25.56, SD = 6.95) and with a mean BMI of 17.58 (SD = 1.64) (see Table 1). Information regarding type of treatment and numbers of years diagnosed for this group is reported in Table 2. An additional 29 remitted anorexics aged 18–47 (M = 27.31, SD = 7.12), with a mean BMI of 22.59 (SD = 3.03) were recruited (see Table 1). Information regarding number of years recovered is reported in Table 2. A total sample of 35 female control participants (M_age_ = 27.20, SD = 8.81; M_BMI_ = 24.77, SD = 4.47), were recruited for the healthy control group (see Table 1).

**Table 1:** Mean and standard deviation (in brackets) of demographics and self-report questionnaires scores for AN (*n* = 27), RAN (*n* = 29) and HCs (*n* = 35).

	AN (*n* = 27) M (SD)	RAN (*n* = 29) M (SD)	HCs (*n* = 35) M (SD)	AN vs. RAN *p*	AN vs HCs *P*	RAN vs. HCs *p*
Age (years)	25.56 (6.95)	27.31 (7.12)	27.20 (8.81)	ns	ns	ns
BMI (kg/cm^2^)	17.58 (1.64)	22.59 (3.03)	24.77 (4.47)	< 0.001	< 0.001	0.029
**EDE-Q**						
Restraint (max 6)	4.15 (1.54)	2.14 (1.93)	1.75 (1.68)	< 0.001	< 0.001	ns
Eating concern (max 6)	3.30 (1.39)	1.83 (1.82)	1.13 (1.23)	0.001	< 0.001	ns
Weight concern (max 6)	4.16 (1.35)	2.96 (1.97)	2.45 (1.87)	0.011	< 0.001	ns
Shape concern (max 6)	4.16 (1.33)	3.07 (1.90)	2.77 (1.68)	0.016	0.001	ns
Global score (max 6)	3.95 (1.24)	2.49 (1.77)	2.03 (1.47)	0.001	< 0.001	ns
**MAIA**						
Noticing (max 5)	3.55 (1.01)	3.31 (1.06)	3.34 (1.07)	ns	ns	ns
Not distracting (max 5)	2.58 (1.14)	2.41 (1.23)	2.80 (0.96)	ns	ns	ns
Not worrying (max 5)	2.85 (1.29)	3.05 (1.16)	2.71 (0.99)	ns	ns	ns
Attention regulation (max 5)	2.80 (0.96)	2.85 (1.20)	2.91 (0.98)	ns	ns	ns
Emotional awareness (max 5)	3.24 (1.30)	3.32 (1.09)	3.46 (1.08)	ns	ns	ns
Self-regulation (max 5)	2.04 (1.25)	2.36 (1.38)	2.83 (1.14)	ns	0.012	ns
Body listening (max 5)	2.00 (1.31)	2.65 (1.24)	2.37 (1.27)	ns	ns	ns
Trusting (max 5)	1.46 (1.32)	2.25 (1.61)	3.14 (1.09)	ns	< 0.001	0.011
**TEAQ**						
Friends and family touch (FFT; max 5)	2.68 (0.72)	2.99 (1.30)	3.51 (0.91)	ns	< 0.001	ns
Current intimate touch (CIT; max 5)	2.51 (0.89)	2.72 (0.97)	3.09 (0.90)	ns	0.014	ns
Childhood touch (ChT; max 5)	3.09 (1.13)	3.02 (1.21)	3.85 (0.83)	ns	0.003	0.002
Attitude to self-care (ASC; max 5)	2.85 (1.07)	3.10 (0.87)	3.77 (0.78)	ns	< 0.001	0.002
Attitude to intimate touch (AIT; max 5)	3.33 (1.02)	3.39 (1.08)	3.93 (0.85)	ns	0.14	0.030
Attitude to unfamiliar touch (AUT; max 5)	2.30 (0.78)	2.36 (0.83)	2.78 (0.96)	ns	0.040	ns

*BMI* Body Mass Index, *EDE-Q* Eating Disorder Examination Questionnaire, *MAIA* Multidimensional Assessment of Interoceptive Awareness, *TEAQ* Touch Experiences and Attitudes Questionnaire, *ns* not significant.

**Table 2:** Demographic characteristics of current anorexics (*n* = 27) recovered anorexics (*n* = 29) and HCs (*n* = 35), which have been analysed by Chi-square.

	AN *n* (%)	RAN *n* (%)	HCs *n* (%)
**Ethnicity**			
Caucasian	21 (77.8%)	24 (82.8%)	29 (82.9%)
Mixed	4 (14.8%)	2 (6.9%)	2 (5.7%)
Black	1 (3.7%)	2 (6.9%)	1 (2.9%)
Asian	1 (3.7%)	1 (3.4%)	3 (8.6%)
**Relationship status**			
Single	11 (40.7%)	14 (48.3%)	14 (40%)
In a relationship	10 (37%)	10 (34.5%)	10 (28.6%)
Engaged	1 (3.7%)	0 (0%)	1 (2.9%)
Married	3 (11.1%)	4 (13.8%)	8 (22.9%)
Separated	0 (0%)	0 (0%)	1 (2.9%)
Divorced	0 (0%)	1 (3.4%)	0 (0%)
Widowed	1 (3.7%)	0 (0%)	0 (0%)
Not specified	0 (0%)	0 (0%)	1 (2.9%)
Prefer not to say	1 (3.7%)	0 (0%)	0 (0%)
**Education level**			
High school	9 (33.3%)	6 (20.7%)	7 (20%)
College	4 (14.8%)	5 (17.2%)	8 (22.9%)
Foundation	3 (11.1%)	1 (3.4%)	2 (5.7%)
Bachelors	6 (22.2%)	9 (31%)	11 (31.4%)
Masters	3 (11.1%)	5 (17.2%)	6 (17.1%)
Doctoral/professional	2 (7.4%)	3 (10.3%)	1 (2.9%)
**Treatment**			
Medication	3 (11.1%)	–	–
Fluoxetine	1 (3.7%)	–	–
SSRI	2 (7.4%)	–	–
Bupropion	1 (3.7%)	–	–
Therapy	6 (22.2%)	–	–
CBT	1 (3.7%)	–	–
Online Therapy	1 (3.7%)	–	–
Therapy not specified	2 (7.4%)	–	–
Psychological/psychiatric treatment	2 (7.4%)	–	–
Day patient treatment	1 (3.7%)	–	–
Medication and therapy	3 (11.1%)	–	–
No treatment	12 (44.4%)	–	–

	AN M (SD)	RAN M (SD)	HCs M (SD)
Number of years diagnosed	6.52 (8.62)	–	–
Number of years recovered	–	4.26 (3.60)	–

Participants for the healthy control group were recruited through external contacts, via social media, the University SONA participant recruitment scheme and through those known to the researchers. Healthy controls were eligible to take part if they self-reported no history or current diagnosis of any psychiatric or neurological disorders (including eating disorders and body dysmorphic disorder) when questioned. No other psychometric constraints were applied because we wanted to recruit women who are representative of the non-eating disordered female population, many of whom have concerns about body image^62^.

Current and remitted AN were recruited by contacting eating disorder charities such as the Beating Eating Disorders Organisation (BEAT) and MQ Mental Health Research, in addition to contacting clinicians known to the researchers, who work directly with these patient groups. Gatekeeper permission was granted to assist with the recruitment of individuals with and recovered from AN. As well as this, patient groups were also recruited through Anorexia support groups on social media. All patients were eligible if they had a primary diagnosis of AN according to the DSM-5^4^. Furthermore, we used scores obtained from the Eating Disorder Examination Questionnaire (EDE-Q) which is a self-report version of the Eating Disorder Examination (EDE) structured interview^63^, as a screening questionnaire for eating disordered behaviour.

All participants had normal or corrected to normal vision (individuals with contact lenses or glasses were able to take part), were not pregnant, did not suffer from any skin conditions such as eczema or any chronic pain conditions such as fibromyalgia. Eligibility for all participants was checked through an online participant screening questionnaire. All participants gave their implied informed consent to take part in the study and were debriefed through the debrief sheet presented at the end of the study. Participants were offered compensation for their time taking part. They were offered the chance to voluntarily enter themselves into a prize draw, with two chances of winning a £25 Amazon voucher and level 4 BSc Psychology students were awarded with course credits. The study was carried out in accordance with the Helsinki declaration of ethical standards. The study protocol was approved by Liverpool John Moores University (LJMU)’s University Research Ethics Committee (UREC, protocol: 20/NSP/025).

## Measures

### Self-report questionnaires

#### Participant screening

An initial set of seven screening questions were used to determine participants’ eligibility. The questionnaire asked participants to answer “true” or “false” to a series of statements. These statements included: “I am 18 years old or over.”, “I am female.”, “I do not suffer from any form of skin condition.”, “I do not suffer from any chronic pain condition.”, “I am not pregnant.”, “I have normal or corrected to normal vision”. A final question asked participants to confirm they had read the participant information sheet and they agreed to take part in the study by clicking an “I agree” option. For the healthy control group only, an additional question ascertained that they did not have a current or previous diagnosis of AN. If a participant answered “false” to any of the statements, or did not agree to take part, an “if then” function was applied, so that ineligible participants were directed to the end of the survey, thanking them for their time in taking part.

To classify clinical populations into AN and RAN groups, patients were required to answer to the following statements: ‘Please specify your current diagnosis of Anorexia Nervosa’ with the options of identifying either: ‘Current diagnosis of Anorexia Nervosa’, ‘Previous diagnosis of Anorexia Nervosa’ or ‘No diagnosis/history of Anorexia Nervosa’. For AN, this group was asked to specify how many years they have had a formal diagnosis, and to declare any treatment they are currently undergoing, through answering the following questions: ‘If you have a current diagnosis of Anorexia Nervosa, for how many years have you had this?’ and ‘Are you currently undergoing any treatment for Anorexia Nervosa (e.g., psychiatric treatments, SSRIs, tranquilizers, and or CBT)?’. The RAN group were asked to identify the number of years they have been recovered from AN: ‘If you have a previous diagnosis of Anorexia Nervosa, for how many years have you been recovered?’. Questions specific to both AN populations were not displayed to individuals with no AN diagnosis.

For both AN and RAN, these groups were required to state whether they had any neurological or psychiatric disorders (as well as Autism Spectrum Disorder, ASD) and to specify the disorder if they did. Three of our AN groups also self-reported that they had comorbidity with ASD. For the healthy control group, answering true to this question would automatically exclude them to ensure they were to be classified in the control group.

### Demographics questionnaire

The demographic information taken from participants included their age, gender, biological sex, ethnicity, relationship status and education level. Participants were also asked to state their height (cm/ft) and weight (Kg/lbs) which was used to calculate their BMI.

### Eating disorder examination questionnaire (EDE-Q)

The Eating Disorder Examination Questionnaire (EDE-Q)^63^ consists of 28 items, which asked participants to self-report any eating disorder symptoms they may have experienced over the last 4 weeks. This questionnaire comprised of four subscales: Restraint, Eating Concern, Weight Concern and Shape Concern. Participants responded to items using a 7-point Likert scale, for the first 12 questions using a scale of 0 = No Days, 6 = Every day. Items for these questions included: “Have you had a definite desire to have a totally flat stomach?” and “Have you had a definite fear that you might gain weight?”. For the next 6 questions participants had to state the number of days (up to 28 days) that each statement corresponded to, statements included: “Over the past 28 days, how many times have you eaten what other people would regard as an usually large amount of food (given the circumstances)?” and Over the past 28 days, how many times have you made yourself sick (vomit) as a means of controlling your shape or weight?”. For the next 6 questions, the scale used ranged from 0 = Not at all to 6 = Markedly, items for these questions included “Has your weight influenced how you think about (judge) yourself as a person?” and “How dissatisfied have you been with your shape?”. The final 4 questions asked participants to self-report their current weight, height, if they have missed any menstrual periods and how many and if they are taking the pill. A sum of scores from all four subscales gave an indication of the severity of eating disorder symptoms. This questionnaire has good internal consistency in clinical populations, with Cronbach α = 0.70–0.83 and in healthy populations. with Cronbach α = 0.78–0.93^64,65^. Individual subscales have also shown to have good internal consistency: Restraint (Cronbach α = 0.70–0.85), Eating Concern (Cronbach α = 0.73–0.86), Shape Concern (Cronbach α = 0.83–0.93), and Weight Concern (Cronbach α = 0.72–0.89)^66^. This questionnaire was administered to assess eating disorder diagnosis and symptom severity.

### Multidimensional assessment of interoceptive awareness questionnaire

The Multidimensional Assessment of Interoceptive Awareness (MAIA)^50^ is a 32‐item questionnaire which was administered to investigate eight dimensions of interoceptive bodily awareness: Noticing (4 items), Not Distracting (3 items), Not Worrying (3 items), Attention Regulation (7 items), Emotional Awareness (5 items), Self-regulation (4 items), Body Listening (3 items) and Trusting (3 items). Items were answered using a 6-point Likert scale ranging from 0 = Never to 5 = Always. Questions included: “When I am tense I notice where the tension is located in my body.” and “I notice when I am uncomfortable in my body”. Each individual dimension is scored by the average of scores from questions corresponding to that subscale, with some questions being reversed scored. This questionnaire is a reliable measure, as it has been previously used in research measuring interoceptive awareness in both healthy^50^ and with an eating disorder population^51^. The MAIA questionnaire was found to have good internal consistency, with Cronbach α = 0.90^67^. For this investigation, we were particularly interested on the MAIA Trusting subscale because previous studies by Brown and colleagues^51,52^ reported that this subscale is the most central in bridging interoceptive awareness and ED symptoms.

### Touch experiences and attitudes questionnaire

The Touch Experiences and Attitudes Questionnaire (TEAQ)^53^ is a 57-item questionnaire which was administered to examine current experiences of positive touch and positive experience of touch during childhood, as well as an individual’s attitude towards positive touch. Questions were answered using a 5-point Likert scale ranging from 1 = “Disagree strongly”, 2 = “Disagree a little”, 3 = “Neither agree nor disagree”, 4 = “Agree a little”, 5 = “Agree strongly”. Questions included: “I dislike people being very physically affectionate towards me.” and “There was a lot of physical affection during my childhood.” A mean score was calculated for each of the six subscales; friends and family touch (FFT) (11 items), current intimate touch (CIT) (14 items), childhood touch (ChT) (9 items), attitude to self-care (ASC) (5 items), attitude to intimate touch (AIT) (13 items) and attitude to unfamiliar touch (AUT) (5 items), with negatively worded questions reversed scored. The TEAQ questionnaire was found to have a good internal consistency with Cronbach α = 0.78–0.92^53,68^. For this investigation, we were particularly interested on the TEAQ Attitude to intimate touch (AIT) subscale because previous investigations suggest that AN patients experience intimate stimuli with lower valence and dominance than healthy controls^54^. They also report difficulties experiencing closeness with a partner and low levels of satisfaction in relationships^55^, they show a fear of intimacy both romantically and sexually^56,57^ and report avoidance of interpersonal relationships with m^58^.

### Self- and other-directed affective touch video clips

**Figure 1:**
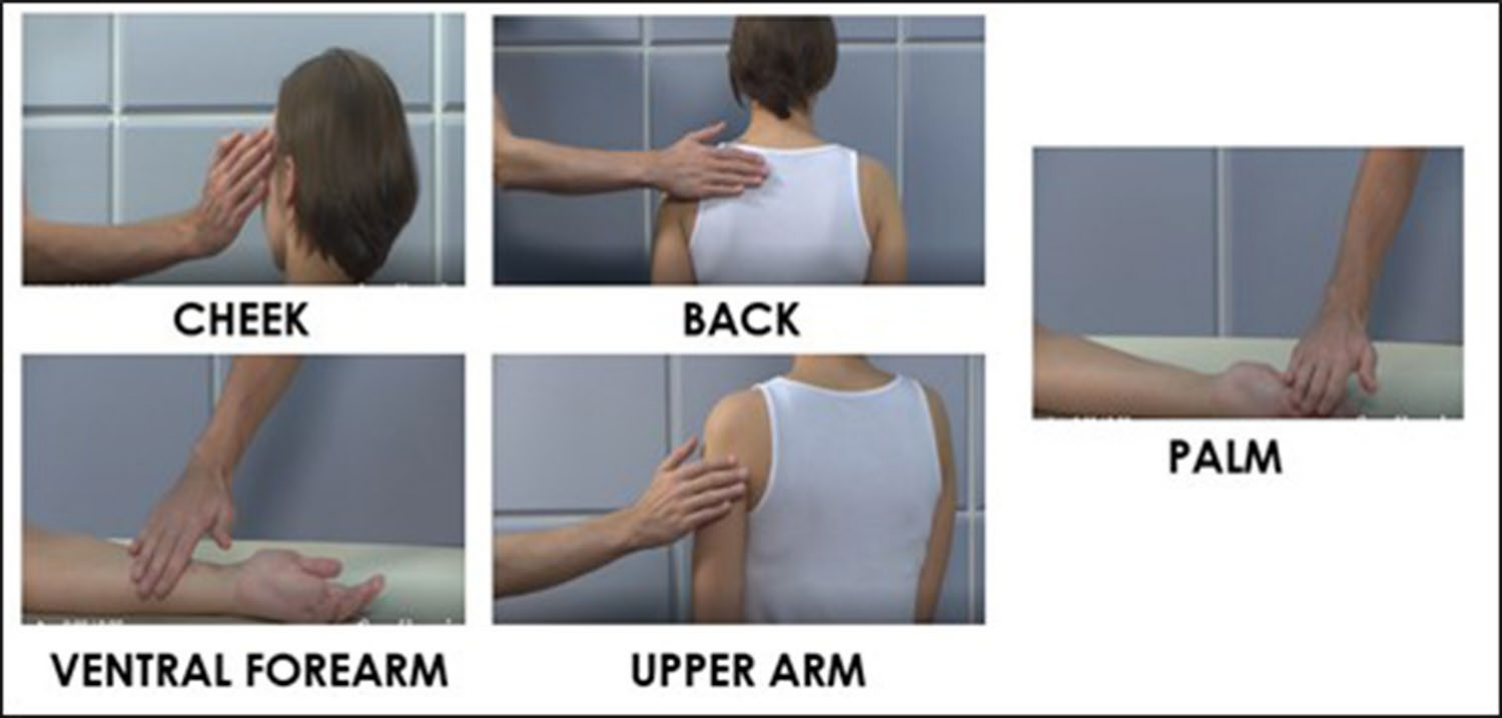
Visual illustration of the 5 body sites (CT-innervated body regions: Ventral Forearm, Upper Arm, Cheek and Back vs. the non-CT innervated palm) from the affective touch videos used in this study.

Affective touch task consisted of 15, 6 s long video clips depicting actors being touched by another person, at different body sites. The videos displayed a female actor receiving touch by a male actor. This choice was made because only females were recruited in the current investigation and we wanted to avoid any confound due to using different genders receiving touch, to those observing this touch. Keeping to females being those receiving touch would make it easier for the female sample to put themselves into the ‘shoes’ of the actor receiving touch. Females tend to be more accurate in perspective taking when the other individual is also a female. For example, in Wacker et al.^69^, mindreading was measured using the Movie for Assessment of Social Cognition, which investigates cognitive and affective mental states including perspective taking and ToM. In this study, females watched a short movie clip and were asked to state thoughts, intentions, and emotions of another actor such as what the actor is feeling. Findings revealed that females were more accurate when the actor was also a female compared to when the actor was a male^69^. Moreover, we chose to display videos showing only males delivering touch and females receiving touch as generally males appear to initiate touch more than females do^70,71^. Furthermore, previous research has found that touch given from an individual of the same sex is generally perceived as less pleasant, compared to touch from an individual of the opposite sex^72^. All videos were taken from a database of previously standardised video clips^68^. Touch was delivered across five different body regions: non-CT-innervated body site i.e., palm vs. CT-innervated body sites i.e., back, ventral forearm, cheek and upper arm) with 3 different stroking velocities: static (0 cm/s), slow (5 cm/s) and fast (30 cm/s). The order in which the videos were presented was counterbalanced amongst participants. To avoid any confound linked to emotional expression of the agent delivering the touch^73^, all videos were displayed zoomed up to the body part of the receiver and the hand of the deliverer of touch, which ensured that the actors faces were not included in the video (see Fig. 1).

Immediately after viewing each video, participants were probed to respond to two questions, one asking: “How much would you like to be touched like that?” (self-directed touch) using a VAS scale ranging from 0 = not at all to 100 = extremely. This question was related to the participant themselves in their level of wanting the touch they are viewing. Participants also responded to a second question asking: “How pleasant do you think that action was for the person being touched?” (3rd person perspective) using a 100-point VAS scale ranging from 0 = very unpleasant to 100 = extremely pleasant^68^. This question concerns the ability of participants to determine mental states of another and evaluate how pleasant the touch received was for the actor in the video (other-directed touch). Overall, there was a total of 15 videos randomly presented, with each video being presented once to participants, each displayed in 240 p YouTube quality in full HD (1920 × 1080 pixels) at 25 fps rate^68^.

### General procedure

The study was conducted online using Qualtrics software, Version 60,939 of the Qualtrics Research Suite (Copyright © 2015 Qualtrics., Provo, UT, USA. http://www.qualtrics.com). Participants were provided with an invitation email containing a brief description of the study and the hyperlink to the online study if participants were happy to take part. Once clicked, participants were provided with the participant information sheet, screening questionnaire and gave their implied consent to take part. Once consent was obtained, participants engaged with the affective touch videos task, during which they were asked to rate using a 100-point VAS scale: “How pleasant do you think that action was for the person being touched?” and “How much would you like to be touched like that?” using a VAS scale ranging from 0 = not at all to 100 = extremely. The order participants viewed the videos was counterbalanced, as well as the order they were shown the two questions. After completion of the videos, participants filled out questionnaires which assessed body image and eating concerns, body awareness, experiences, and attitudes to touch, as well as social isolation and longing for touch since the COVID-19 pandemic respectively, by means of the EDE-Q^63^, MAIA^50^, TEAQ^53^ and the COVID-19 touch experiences and eating behaviour Questionnaire (see Supplementary Information). The order participants filled out these questionnaires was counterbalanced. Those participants who were not eligible were taken to the end of the survey, in which they were thanked for their time. Overall, the online study lasted approximately 35 min. Data collection began on 10th July 2020 and ended on 27th June 2021.

### Data handling and statistical analysis

Data were analysed using STATISTICA 8.0 (StatSoftInc, Tulsa, Oklahoma). Inspection of model residuals indicated data were normally distributed. Assumptions of sphericity were not violated. Third-party pleasantness ratings of social touch were analysed by conducting a 4-way Mixed ANOVA with a between-subjects factor of Group (3 levels: AN, RAN and HCs) and within-subjects factors of velocity (3 levels: 0 cm/s, 5 cm/s and 30 cm/s), body sites (5 levels: Upper arm, ventral forearm, back, cheek and palm) and Task (2 levels: Other-directed touch and self-directed touch). All demographic information and scores for the self-reports are reported as Mean (M) and Standard Deviation of the mean (SD). A significance threshold of *p* < 0.05 was used as the significance threshold for each of the effects. All pairwise comparisons were assessed using the Duncan's post-hoc test correction for multiple comparisons, which reduces the size of the critical difference depending on the number of steps separating the ordered means. This procedure is optimal for testing in the same design effects that may have different sizes^74–76^, as it is expected in our case for differences between patients AN, RAN and HCs participants and, within the patient group, between AN and RAN patients, as well as for the size of the effects of the different within-subject variables (i.e., Self- vs. Other-directed touch effects).

Independent samples t-tests, with Bonferroni Correction were conducted to assess group differences for subscales of the EDE-Q, MAIA and TEAQ questionnaires, by examining significant differences in mean scores across each group. A significance threshold of *p* < 0.05 was used as the significance threshold for each of the effects. For exploratory purposes we considered all 4 factors of the EDE-Q, to also take into account those factors which focus on the importance of and preoccupation with shape and weight, which the 3-factor model does not include^65^.

In keeping with previous studies^77,78^, and due to our findings suggesting that reduced pleasantness of self-directed touch was CT-optimal specific (please see Self-directed vs Other-directed affective touch ratings for full results), a Pleasant Touch Awareness (PTA) index was calculated as the difference in pleasantness ratings between CT-optimal (5 cm/s) and CT non-optimal stroking (30 cm/s), weighted by the average scores calculated separately for each participant and location (PTA = (pleasantness ratings at 5 cm/s − pleasantness ratings at 30 cm/s)/overall touch pleasantness). PTA measures the degree to which an individual prefers CT-optimal as opposed to CT non-optimal velocities^79^, and it was used in the current study to compare preference for CT-optimal touch across the three groups (AN, RAN and HCs) and the two tasks. Furthermore, an Overall Touch Pleasantness (OTP index was calculated as the average across CT-optimal velocity (5 cm/s) and CT-non optimal velocities (0 cm/s and 30 cm/s). This index was calculated separately for each group and task^77,78^. OTP refers to the extent to which an individual finds touch pleasant, which is not CT-specific. Thus, in the current study we wanted to examine overall touch pleasantness amongst the three groups (AN, RAN and HCs) and self- vs. other-directed social touch.

For exploratory purposes, we performed a series of exploratory Multiple Linear Regression analyses to investigate the predictive role of TEAQ AIT and MAIA Trusting subscales on both PTA and OTA, across the three groups and tasks.

## Results

### Univariate statistics

Table 1 reports means and standard deviations for the demographic and psychometric questionnaire subscales, which has been calculated separately for AN, RAN and HCs. The far-right column shows the output of pairwise comparisons between the three groups, which have been adjusted for multiple comparisons (Bonferroni-corrected). The three groups were matched for age. However, as expected, all groups differed regarding their self-reported BMI, with AN having a significantly lower BMI than HCs and RAN, and RAN had significantly lower (but still in the normal-weight range) BMI than HCs. Furthermore, as expected, AN had significantly higher EDE-Q global scores than RAN and HCs, whilst no difference was observed between RAN and HCs.

Between AN and HCs, there was a significant difference observed for the subscale MAIA Self-regulation, with HCs demonstrating higher ability to self-regulate distress when paying attention to inner body sensations, compared to AN. HCs had also significantly higher MAIA Trusting scores and thus reported greater experiences of their body as safe and trustworthy compared to both AN and RAN. No other group differences were observed for any other interoceptive facet, as measured by the MAIA.

For touch experiences and attitudes, HCs currently experience more Family and Friends touch and current intimate touch compared to AN. During childhood, HCs reported experiencing significantly more childhood touch compared to both AN and RAN. HCs also reported more positive attitudes towards self-care, and intimate touch compared to both AN and RAN and reported higher attitudes to unfamiliar touch compared to AN. Both AN and RAN did not differ in their current and previous touch experiences, and both demonstrated similar attitudes towards touch and self-care.

Additional demographics such as ethnicity, relationship status and education level were also collected and are reported in Table 2. We carried out Chi-Square analysis between all 3 groups to investigate whether there were any differences in these demographics between groups. Importantly, there was no significant differences in ethnicity (χ^2^_6_ = 3.28; *p* = 0.773), relationship status (χ^2^_16_ = 13.18; *p* = 0.659) and education level (χ^2^_10_ = 5.33; *p* = 0.868) between groups.

### Self-directed vs other-directed affective touch ratings

The repeated measures ANOVA revealed a significant main effect of Body sites [*F*(4,352) = 19.10, *p* < 0.001, *ηp*^2^ = 0.18], with the back being rated as most pleasant overall (all *ps* < 0.001). The upper arm, ventral forearm and palm were all rated as more pleasant than the cheek (all *ps* < 0.001). No other significant differences in pleasant ratings for the other body sites were observed (all *ps* > *0.351*). There was also a significant main effect of velocity [*F*(2,176) = 11.99, *p* < 0.001, *ηp*^2^ = 0.12], with CT-optimal touch (5 cm/s) rated as significantly more pleasant than touch applied at CT non-optimal velocities (0cms/s and 30 cm/s) (all *ps* < 0.001). No significant differences were observed between the two CT non-optimal velocities (*p* = 0.682). There was a significant main effect of Task [*F*(1,88) = 67.57, *p* < 0.001, *ηp*^2^ = 0.43], with overall touch being rated as more pleasant for other-directed touch compared to self-directed touch (*p* < 0.001). A significant main effect of Group was also observed [*F*(2, 88) = 6.43, *p* = 0.002, *ηp*^2^ = 0.13]. Overall, there were significant differences in pleasantness ratings between both HCs and AN (*p* = 0.022) and HCs and RAN (*p* = 0.002), with HCs providing higher overall pleasantness ratings compared to AN and RAN. No significant differences were observed in pleasantness ratings provided by AN and RAN (*p* = 0.301).

A significant 2-way interaction of body sites × velocity was revealed [*F* (8, 704) = 8.64, *p* < 0*.*001, *ηp*^2^ = 0.09] with a velocity of 5 cm/s to the ventral forearm being rated as significantly more pleasant than 30 cm/s to the ventral forearm (*p* < 0.001) and 0 cm/s to the ventral forearm (*p* < 0.001). Touch delivered at 5 cm/s to the cheek was rated as significantly more pleasant than touch delivered at both 30 cm/s (*p* < 0.001) and 0 cm/s (*p* < 0.001). Also, touch delivered at 5 cm/s to the palm was rated as significantly more pleasant than that delivered at 30 cm/s (*p* = 0.007) and 0 cm/s (*p* = 0.005). All other body sites and velocities were not significant (*p* > 0*.*224).

**Figure 2:**
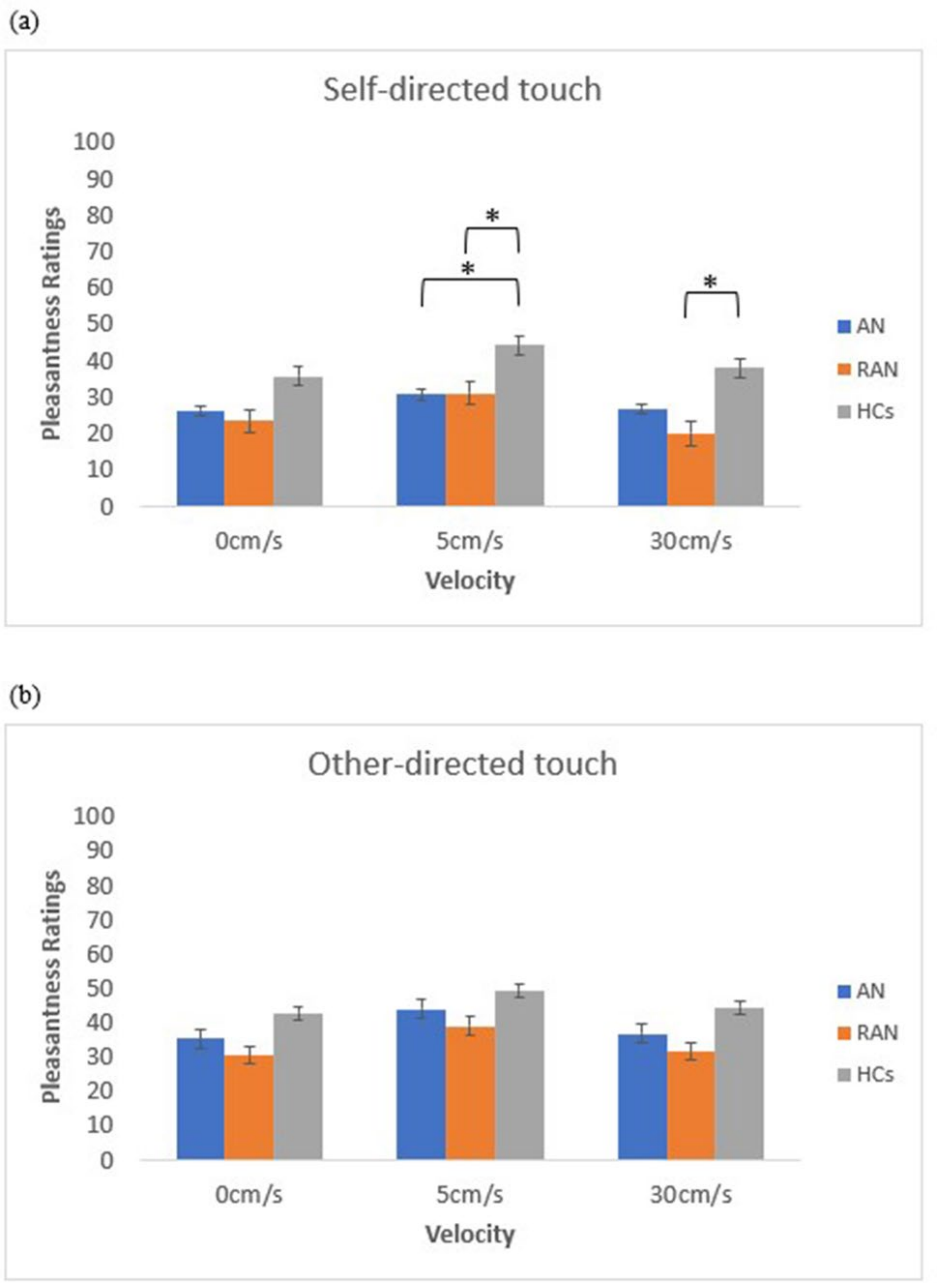
Pleasantness ratings for each velocity, which have been collapsed across body sites, with each bar representing one of three groups (AN, RAN and HCs). Graphs are separated for each task: (**a**) self-directed touch and (**b**) other-directed touch.

Crucially, a 3-way interaction of velocity × task × group was also observed [*F*(4, 176) = 2.99, *p* = 0.020, *ηp*^2^ = 0.06; See Fig. 2]. For self-directed touch, post-hoc comparisons of the three groups of AN, RAN and HCs at each level of velocity revealed that AN and RAN rated touch delivered at 5 cm/s significantly lower than HCs (all *p* < 0.05). No difference in pleasantness ratings was observed between AN and RAN for touch delivered at CT-optimal velocity (*p* = 0.946). A significant difference was also observed between pleasantness ratings provided by HCs and RAN for 30 cm/s (*p* = 0.007), with HCs providing higher preference ratings for 30 cm/s compared to RAN. There were no significant differences amongst the three groups in their ratings of 0 cm/s touch (all *ps* > 0.429). For the other-directed task, there was no significant difference between groups for any of the velocities (*all ps* > 0.05) (For a breakdown of results, please see Table 3).

**Table 3:** The repeated measures ANOVA results for self-directed vs other-directed affective touch ratings, which includes the degrees of freedom, F value, p value and effect size.

Effect	*df*_*1*_	*df*_*2*_	*F*	*p*	*ηp*^2^
Body site	4	352	19.10	< 0.001	0.18
Velocity	2	176	11.99	< 0.001	0.12
Task	1	88	67.57	< 0.001	0.43
Group	2	88	6.43	0.002	0.13
Body site*velocity	8	704	8.64	< 0.001	0.09
Velocity*task*group	4	176	2.99	0.020	0.06

Each main effect and the significant 2-way and 3-way interactions are reported below.

*df* degrees of freedom, *F* variance between sample means/variation within the samples, *p* probability value, *ηp*^2^ effect size.

In summary, regardless of whether touch involved the self or another person, all groups demonstrated a preference for CT-optimal touch (5 cm/s) compared to CT-non optimal touch (0 cm/s and 30 cm/s). For all groups, touch evaluated for another person (other-directed touch) was perceived as more pleasant compared to when participants were asked to judge receiving the same touch to themselves (self-directed touch). Crucially, both AN and RAN demonstrated lower preference for self-directed touch when delivered at CT-optimal velocity (5 cm/s) than HCs. For the other-directed task, there was no significant difference between groups for any of the velocities.

### Pleasant touch awareness and overall touch perception related to attitudes in intimate touch and the trusting of one’s own body

We performed a series of exploratory (given the relatively small sample size for such analyses) Multiple Linear Regression analyses to explore whether individual differences in experiences to intimate touch and of one's body as safe and trustworthy could predict AN, RAN and HCs’ preference for CT-optimal touch (PTA) or for overall touch (regardless of CT-Optimal specificity, OTP). Accordingly, we included TEAQ AIT and MAIA Trusting as predictors of PTA and OTP respectively for self- and other-directed touch. We repeated these regressions for each group separately.

For self-directed touch, we found a significant regression equation for the overall touch pleasantness for HCs [*F*(2,34) = 4.359, *p* = 0.021] and for AN [*F*(2,26) = 6.365, *p* = 0.006]. For RAN, the regression equation was only marginally significant [*F*(2,28) = 3.219, *p* = 0.056]. Specifically, the TEAQ AIT was a significant predictor for overall touch preference for both HCs and AN, suggesting higher positive attitude towards intimate touch to predict higher pleasantness ratings to social touch (regardless of CT optimality of the stimulation) in both HCs and AN, but not in RAN (see Table 4). For other-directed touch, we did not find significant regression equations for the overall touch pleasantness for HCs [*F*(2,34) = 0.839, *p* = 0.441], AN [*F*(2,26) = 1.635, *p* = 0.216] or RAN [*F*(2,28) = 0.638, *p* = 0.537] (See Table 3).

**Table 4:** Unstandardized coefficients of the multiple linear regression model for MAIA Trusting and TEAQ Attitude to intimate touch (AIT) for both PTA and OTP separated for each group (AN, RAN and HCs) and task (self-directed and other-directed touch).

				B	SE	*β*	*t-*value	*p*-level
HCs	Self-directed	PTA	MAIA_trusting	0.040	0.087	0.082	0.464	0.646
			TEAQ_AIT	− 0.011	0.111	− 0.018	− 0.102	0.920
AN			MAIA_trusting	0.063	0.100	0.129	0.624	0.538
			TEAQ_AIT	0.030	0.129	0.048	0.233	0.818
RAN			MAIA_trusting	− 0.002	0.117	− 0.003	− 0.014	0.989
			TEAQ_AIT	− 0.188	0.175	− 0.227	− 01.072	0.293
HCs	Other-directed		MAIA_trusting	0.057	0.062	0.161	0.918	0.365
			TEAQ_AIT	− 0.017	0.079	− 0.037	− 0.214	0.832
AN			MAIA_trusting	0.019	0.066	0.059	0.290	0.775
			TEAQ_AIT	0.061	0.085	0.147	0.718	0.480
RAN			MAIA_trusting	0.033	0.078	0.091	0.422	0.677
			TEAQ_AIT	− 0.020	0.116	− 0.037	− 0.173	0.864
HCs	Self-directed	OTP	MAIA_trusting	1.237	2.416	0.080	0.512	0.612
			TEAQ_AIT	8.798	3.074	0.450	2.862	0.007*
AN			MAIA_trusting	3.135	2.041	0.259	1.536	0.138
			TEAQ_AIT	7.481	2.632	0.479	2.843	0.009*
RAN			MAIA_trusting	4.347	2.078	0.407	2.092	0.046
			TEAQ_AIT	1.218	3.108	0.076	0.392	0.698
HCs	Other-directed		MAIA_trusting	0.349	2.152	0.028	0.162	0.872
			TEAQ_AIT	3.477	2.738	0.219	1.270	0.213
AN			MAIA_trusting	2.126	2.239	0.186	0.950	0.352
			TEAQ_AIT	3.796	2.887	0.257	1.315	0.201
RAN			MAIA_trusting	2.345	2.215	0.225	1.059	0.299
			TEAQ_AIT	− 0.341	3.314	− 0.022	− 0.103	0.919

*AN* Current Anorexics, *RAN* Remitted Anorexics, *HCs* Healthy Controls, *PTA* Pleasant Touch Awareness, *OTP* Overall Touch Pleasantness, *MAIA* Multidimensional Assessment of Interoceptive Awareness, *TEAQ* Touch Experiences and Attitudes Questionnaire, *AIT* Attitudes to Intimate Touch. *Indicates p < 0.01.

We then conducted a separate Multiple Linear Regression analysis for each group to investigate whether TEAQ AIT and MAIA Trusting would predict women’ PTA for self-directed touch. Again, we did not find significant regression equations for neither of the three groups (HCs [*F*(2,34) = 0.110, *p* = 0.896], AN [*F*(2,26) = 0.262, *p* = 0.772], RAN [*F*(2,28) = 0.715, *p* = 0.499]). Similarly, we did not find significant regression equations for PTA for other-directed touch in any of the three groups (HCs [*F*(2,34) = 0.432, *p* = 0.653], AN [*F*(2,26) = 0.356, *p* = 0.704] and RAN [*F*(2,28) = 0.089, *p* = 0.915], See Table 4).

## Discussion

To the best of our knowledge, this is the first investigation to assess whether third-party vicarious ratings of self- vs. other-directed social touch, delivered at CT-optimal vs. CT non-optimal velocities differ in women with a current or previous diagnosis of AN, compared to healthy individuals with no AN diagnosis. Our analysis also included covariates such as interoceptive awareness and attitudes to touch with the aim to explore whether potential factors which are known to largely contribute towards the aetiology and development of EDs^1,9–11^ might also explain subjective ratings of pleasantness for social touch.

When taking into consideration both self- and other-directed touch tasks, both groups of patients and healthy comparison subjects showed the expected velocity dependent pleasantness curve for brush stroking, indicating that C-tactile targeted stroking velocities were preferred over slower and faster velocities^20,80^. Although the case, both AN and RAN demonstrated significantly lower pleasantness ratings for CT-optimal touch compared to HCs. This finding is in line with those of real experiences to touch by Crucianelli et al.^14^ as they found that anorexics rated CT-optimal touch (delivered to the self) as less pleasant than healthy controls, which was not evident for CT-non optimal velocities. Additionally, this finding supports research by Davidovic et al.^13^, whereby current anorexics rated touch as less pleasant than matched HCs. Yet, this finding contradicts the results obtained by Crucianelli et al.^15^ as they found that a reduction in tactile pleasantness was not CT-specific. A possible explanation for this finding is that atypical responses to affective touch is due to an error in bottom-up processing, i.e., an error in the sensation and perception of touch, which could, as suggested by Crucianelli et al.^14^, be due to a dysfunctional CT afferent system, particularly as this finding is particularly evident in the self-directed task.

A possible mutual but not exclusive explanation concerning atypical responses to affective touch to the self could be due to anorexics’ anxiety and discomfort towards touch in general^81^. Accordingly, we observed significant differences in the subjective ratings to experience and attitude to social touch across all domains of touch experience and amongst the three groups. Indeed, AN reported significantly less positive experience to familiar and unfamiliar touch, as well as to intimate touch compared to HCs. Interestingly, a significant regression equation for self-reports of touch experiences and overall touch pleasantness for AN, particularly for vicarious self-directed touch lend support to this idea. With these regards, it was found that positive attitude to intimate touch was positively associated with overall touch pleasantness for current anorexics (but also for HCs), when concerning touch to the self. This is in line with previous findings from Zucker et al.^44^, who investigated sensitivity and avoidance of touch in women with current and a previous diagnosis of AN, in comparison to HCs. Both clinical groups also demonstrated higher sensitivity and avoidance of touch compared to HCs, with AN demonstrating higher touch sensitivity. Furthermore, another possible explanation for the association between attitude to intimate touch and touch perception to the self-directed touch is that typically anorexics tend to struggle and find closeness discomforting and have difficulty in maintaining close intimate relationships with a romantic partner, family, and friends^55,81^. The more severe the body image disturbances in these populations, the less likely they are to engage and feel comfortable in physical intimacy i.e., touch from a romantic partner or feel comfortable with touch from a loved one^82^. This could explain the reduction in pleasantness of touch to the self for both current anorexics, as low exposure to touch was found to result in lower pleasantness of touch^83^. Yet, our findings are not conclusive and should be interpreted with caution given they are exploratory in their nature. As such, further studies with larger clinical samples should be conducted to better investigate the contribution of social touch experiences and attitudes in predicting anorexics’ sensitivity to CT-optimal affective touch.

Contrary to our prediction, we did not find evidence that experience of one's body as safe and trustworthy (MAIA Trusting) might predict women’ preference for CT-Optimal touch nor for the overall touch perception, in the three groups. Previous works by Brown et al.^52^ reported that items from the MAIA Trusting subscale were the most central in bridging interoceptive awareness and ED symptoms^52^. However, the study by Crucianelli et al.^15^ reported that individual differences in the perceived pleasantness of CT-optimal in the AN group were predicted by interoceptive sensibility scores (as measured by the Body Awareness Questionnaire^84^, thus suggesting that other facets of interoception awareness might play a role in the perception of social touch in AN. Thus, future studies in AN are needed to disentangle the exact contribution of multidimensions of interoceptive awareness to preference to social touch to draw firm conclusions about the mechanisms that cause blunted responses to vicarious experience of social touch in this population.

Overall, we observed group differences for third party ratings of pleasantness of touch for self but not for another. We capitalized on prior research which has reported that individuals with AN have impaired ToM which involves the decoding of affective stimuli^38^ both at self and other perspectives^36,37^ and it was found to manifest even after recovery^39–44,85^. In this study, it was expected that anorexics would use their own experiences to interpret the feelings generated in another, which would be less pleasant compared to neurotypical populations. However, both current and remitted anorexics did not perceive CT-targeted touch as less pleasant than healthy controls for other-directed touch. Instead, as found previously, no differences were observed between anorexics and control participants regarding perspective taking when engaging in ratings for other-directed touch^38,86–88^. A possible explanation for this finding could be related to ‘learned experiences’ of touch as pleasant when delivered to another, which conversely does not apply to the self. With these regards, it might be the case that anorexics may observe how others respond to the receiving of touch and use this learned experience when making judgments about touch for others, regardless of the fact that they perceive touch to themselves as less pleasant^89^. Accordingly, they may have used their experience of how others evaluate pleasantness of touch or how they physically respond when receiving touch, to guide their interpretation when making inferences about other-directed touch^48^. In doing so, they may have boosted their ToM abilities, which is a process which enhances based on an individual’s experience of that specific interaction. However, another possible but not mutually exclusive explanation is that, like found previously, anorexics have an intact ToM functioning, comparable to healthy controls^38,86–89^ but as suggested by Sedgewick et al.^89^, the social-emotional difficulties anorexics face may not be specific to inferring and recognising emotions and mental states. Rather this would reflect a bias in the interpretation of their social situations that they are more likely to avoid. Furthermore, it also could be that AN struggle with more complex social situations involving a dynamic of various interactions, as opposed to less complex social situations such as receiving pleasant touch from another individual^89^.

Although this study was the first to assess third-party ratings of social touch in current and remitted anorexics, limitations have been identified. First, self-report diagnosis may have posed an issue with the variability of individuals in both clinical samples and also there is a risk of some undiagnosed in the healthy control group. However, these concerns are mitigated by several factors. First, the online survey was anonymous, which should encourage honest responding. Furthermore, participants’ ability to continue to take part to the study depended on their responses, which encouraged the provision of high-quality data. Most importantly, the manipulation check indicated that individuals reporting AN endorsed significantly greater eating pathology on the EDE-Q which is a gold standard measure for the assessment of symptom severity and AN diagnosis in replacement of structured interviews (i.e., Eating disorder examination interview^90,91^. Future studies should however attempt to replicate these findings by recruiting AN inpatients whose diagnosis has been established by a qualified clinician. Although constrained due to the Covid-19 pandemic lockdown, it would have been beneficial to have included a real touch condition, particularly to compare vicarious vs actual self-directed touch, as also conducted by Crucianelli et al.^15^. Future studies should focus on this distinction to clarify whether an impairment of vicarious representation alone as opposed to a combination of both actual and vicarious representations of self-directed affective touch might characterise EDs populations^92^.

Although the study controlled for various variables that may affect touch pleasantness, other factors such as touch avoidance, sexuality, mood and emphatic traits may have an impact as well. For example, a recent study by Sailer and Ackerley^83^ found participants with low touch exposure scores, which on the Social Touch Questionnaire (STQ)^93^ is indicative of unpleasantness of social touch and avoidance, rated touch differently, as they could not differentiate various stroking velocities. Furthermore, mood may have influenced social touch perception. Although participants were asked to state if they had any neurological or psychiatric conditions, a form of anhedonia measurement such as the Fawcett-Clark Pleasure Scale (FCPS)^94^ or the Snaith-Hamilton Pleasure Scale (SHAPS)^95^ should have been administered to account for participants mood on their testing day and those above the cut-off point excluded. This is because anhedonia, a characteristic for depression associated with the reduced ability to experience pleasure, can negatively affect attitudes to social touch^96^. Furthermore, in Crucianelli et al.^15^ study, tactile anhedonia was found to be present in RAN as well as AN, suggesting that this could be a trait which manifests even post-recovery which could account for their lack of pleasure towards touch.

Another factor which may have impacted the third-party vicarious ratings of social touch is individual differences in empathic abilities. This factor influences participants’ ability during embodiment tasks and vicarious experiences^97–99^. For example, Peled-Avron et al.^100^ found levels of empathy modified ratings and EEG responses to photos of social touch. Participants classified as highly empathetic, had higher mu suppression and rated social touch as more pleasant, as opposed to participants reporting lower empathy levels. Therefore, it would be prudent in future studies that involve third-party ratings of touch, rather than actual touch, to consider empathy measures, as responses to observed touch are modulated by empathy levels and previous research has found that current anorexics have lower levels of empathy compared to RAN and HCs^41,101^.

## Conclusions

To conclude, our findings add to previous results of Crucianelli et al.^14^ and Davidovic and colleagues^13^ in that they show that despite atypical, blunted responses to affective touch, women with AN and RAN still demonstrate preference for CT-optimal touch. Most importantly, we provide initial evidence that third-party ratings of touch pleasantness for another individual do not differ regardless of ED diagnosis. On the contrary, both RAN and AN groups rated affective touch as less pleasant compared to healthy controls when touch involved the self. Thus, altered responses to vicarious experience of affective touch in AN and RAN might be self-specific and intact when making judgements of touch for someone else. This could be due to a learning mechanism according to which AN have learned that the touch they are viewing would be pleasant for another individual, with multiple facets of interoceptive awareness and social touch experiences possibly mediating this effect. Given that atypical responses persist even after recovery, treatment interventions should focus on helping anorexics to better interpret or improve tolerance of affective tactile experiences when self-directed. Overcoming the impairment in responses to self-directed affective touch could help prevent post-recovery relapsing, particularly as previous research has suggested that this impairment stems from their distorted mental imagery i.e., body image distortions and distorted beliefs, which are key characteristics in the maintenance of this disorder. For example, mirror therapy, which involves the repetitive exposure of oneself in a mirror performing a specific behaviour^102^, can be integrated with self-touch to improve body awareness and decrease negative response to vision of the body^103^. This can be achieved through the repetitive action of performing self-touch to different body regions whilst constantly being exposed to a visual depiction of the body.

## Supplementary Information

Supplementary Information.

## Data Availability

The data that support the findings of this study are available on request from the corresponding author. The data are not publicly available due to privacy or ethical restrictions.
