# Supplementary material for: Vicarious ratings of self vs. other-directed social touch in women with and recovered from Anorexia Nervosa
Source: Sci Rep. 2022 Aug 4;12:13429. doi: 10.1038/s41598-022-17523-2 (PMC9352775; doi:10.1038/s41598-022-17523-2)
Supplement: Supplementary file 1 — Supplementary Information. [file 41598_2022_17523_MOESM1_ESM.pdf]

Vicarious Ratings of Self vs. Other-directed Social Touch in Women with and Recovered from Anorexia Nervosa

Ashleigh Bellard[1*], Paula Trotter[1], Francis McGlone[1] & Valentina Cazzato[1]

[1]School of Psychology, Faculty of Health, Liverpool John Moores University,

Liverpool, UK

Supplementary Information 1

Pleasant touch awareness and overall touch perception related to touch experiences and attitudes and interoceptive awareness

For exploratory purposes, we then conducted Pearson's correlations with a Bonferroni correction amongst all self-report subscales to assess any association between MAIA and TEAQ questionnaires with the PTA and OTP indices, separately for each group.

Pleasant Touch Awareness Correlations

We wanted, for exploratory purposes to investigate whether individual differences in interoceptive awareness and touch experiences and attitudes, as measured by the MAIA and TEAQ respectively, were associated with pleasant touch awareness index (PTA) for each group, calculated separately per task.

For self-directed touch, PTA was negatively associated with MAIA-Noticing (r= -.516, p= .006) for HCs. In other words, for HCs, the ability to notice both comfortable and uncomfortable sensations in the body was negatively associated with PTA. Thus, the higher the ability to notice both comfortable and uncomfortable inner body sensations, the lower their sensitivity to CT-optimal touch. For both AN and RAN, no subscales predicted PTA index for these groups.

For other-directed touch, PTA was negatively correlated with MAIA-Noticing (*r*= -.437, *p*= .009) and MAIA-EA (*r*= -.436, *p*= .009) for HCs. For HCs awareness of comfortable and uncomfortable sensations and emotional awareness was negatively associated with PTA. Thus, for HCs, the higher the PTA the lower the ability to notice comfortable and uncomfortable sensations in the body. For AN, no subscales predicted positive touch awareness for this group. For RAN, MAIA-Not Worrying was positively associated with positive touch awareness (*r*= .446, *p*= .015). The ability to not experience distress when experiencing negative sensations in the body was positively associated with greater sensitivity to CT-optimal touch for RAN.

To sum, pleasantness of CT-optimal touch involving the self and touch to another was explained by one's lack of ability to notice inner bodily sensations, both comfortable and uncomfortable for HCs only. Evaluating pleasantness of CT-optimal touch involving another person was explained by one's ability to not distress when experiencing negative inner bodily sensations.

For all groups, PTA for self- and other-directed touch did not correlate with any of the TEAQ subscales.

Overall Touch Pleasantness Correlations

We also wanted, for exploratory purposes, to investigate whether individual differences in interoceptive awareness and touch experiences and attitudes, as measured by the MAIA and TEAQ, were associated with overall touch pleasantness for each group, calculated separately per task.

For self-directed touch, OTP was positively associated with TEAQ-AIT (*r*= .456, *p*= .006) and TEAQ-AUT (*r*= .485, *p*= .003) for HCs. Specifically, positive attitudes towards touch from both an intimate partner and from unfamiliar touch were associated with overall touch pleasantness for this group, as both these predictors increased, so did overall touch pleasantness. For AN, OTP was positively associated with MAIA_Body Listening (*r*= .402, *p*=. 038). In addition, current intimate touch and attitudes were positively associated with overall touch pleasantness (TEAQ-CIT: *r*= .566, *p*= .002; TEAQ-AIT: *r*= .531, *p*= .004). For RAN, MAIA-BL (*r*= .527, *p*= .003), MAIA-SR (*r*= .461, *p*= .012), MAIA-EA (*r*= .695, *p*<.001), MAIA-AR (*r*= .612, *p*<.001) were positively associated with OTP and TEAQ-CIT

(*r*= -.465, *p*= .011) was negatively associated with OTP. In other words, for the RAN group, listening to one's body, ability to regulate distress from one's own body, emotional awareness and attention regulation were all positively associated with higher overall touch pleasantness. However, current intimate touch was negatively associated with overall touch pleasantness. As intimate touch increases, overall touch pleasantness decreases (see Table S1, in Supplementary Information).

For other-directed touch, no subscale was significantly associated with OTP for any of the groups (see Table S1, in Supplementary Information).

To sum, pleasantness of touch involving the self was explained by more positive attitudes towards intimate and unfamiliar touch for HCs. For AN, receiving more intimate touch and having positive attitudes towards this intimate touch explained greater pleasantness for touch (regardless of velocity). Pleasantness of touch for RAN was greater due to interoceptive awareness ability i.e., their capacity to listen and pay more attention to sensations in the body, regulate distress and the ability to understand internal emotional sensations of the body. The evaluation of pleasantness of touch for another person was not explained by any factors involving touch experiences and attitudes or interoceptive facets for any group.

Table S1. Pearson's correlational analysis between OTP and subscales from the DCQ, MAIA and TEAQ Questionnaires. Correlations have been performed separately for each group, AN (*n*= *27*), RAN (*n*= *29*) and HCs (*n*= *35*).

			1	2	3	4	5	6	7	8	9	10	11	12	13	15	15	16
AN	1.	OTP_ Other-directed	-	.813**	-.005	.162	.110	.080	.164	.151	.184	.238	.151	.415*	-.126	.148	.295	-.246
	2.	OTP_ Self-directed		-	-.181	.267	.075	.197	.209	.225	.402*	.356	.400*	.566**	-.025	.261	.531**	-.049
	3.	MAIA_ Noticing			-	-.064	-.510**	.287	.517**	.125	.073	-.067	.192	.127	.059	.343	.298	-.195
	4.	MAIA_ Not Distracting				-	-.382*	-.216	.194	.074	.287	.178	.485*	.407	.359	.348	.468*	-.060
	5.	MAIA_ Not worrying					-	-.166	-.420*	-.068	-.301	-.328	-.431*	-.269	-.142	-.487*	-.381*	-.141
	6.	MAIA_ Attention Regulation						-	.313	.450*	.375	.489**	.308	.322	-.064	.355	.221	.071

	7.	MAIA _ Emotional Awareness							-	.105	.471*	.160	.208	.391*	-.123	.213	.463*	.253
	8.	MAIA _ Self Regulation								-	.504**	.442*	.495**	.065	.039	.375	.187	-.123
	9.	MAIA _ Body Listening									-	.499**	.393*	.555**	.070	.520**	.267	.138
	10.	MAIA _ Trusting										-	.465*	.351	.080	.444*	.202	.114
	11.	TEAQ _ FFT											-	.407*	.386*	.571**	.436*	.039
	12.	TEAQ _ CIT												-	.132	.357	.535**	-.149
	13.	TEAQ _ ChT													-	.188	-.023	-.007
	14.	TEAQ _ ASC														-	.263	-.052
	15.	TEAQ _ AIT															-	-.084
	16.	TEAQ _ AUT																-
RAN	1.	OTP_ Other-directed	-	.812**	-.357	-.247	.257	-.027	-.065	.121	.223	.215	-.140	-.465*	.016	-.349	.075	.071
	2.	OTP_ Self-directed		-	-.095	-.102	.064	.175	.169	.255	.404*	.440*	-.072	-.220	.033	-.269	.252	.363
	3.	MAIA _ Noticing			-	-.250	-.331	.612**	.695**	.461*	.527**	.381*	.049	-.003	-.092	.060	.174	-.079
	4.	MAIA _ Not Distracting				-	-.231	-.289	-.132	-.103	-.162	-.090	.272	.203	.196	.144	-.091	.380*
	5.	MAIA _ Not worrying					-	-.264	-.451*	-.254	-.349	-.205	-.121	-.413*	.174	-.220	-.209	.007
	6.	MAIA _ Attention Regulation						-	.633**	.634**	.680**	.568**	.245	.069	.240	.180	.208	.053
	7.	MAIA _ Emotional Awareness							-	.589**	.638**	.530**	-.014	.112	-.103	.009	.143	-.047
	8.	MAIA _ Self Regulation								-	.833**	.855**	.279	.083	.042	-.126	.342	.169
	9.	MAIA _ Body Listening									-	.808**	.329	-.015	.147	-.256	.325	.078
	10.	MAIA _ Trusting										-	.258	.113	.212	-.145	.432*	.239
	11.	TEAQ _ FFT											-	.425*	.583**	.351	.376*	.385*

	12.	TEAQ_CIT												-	.243	.488**	.562**	.364
	13.	TEAQ_ChT													-	.229	.237	.358
	14.	TEAQ_ASC														-	.250	.261
	15.	TEAQ_AIT															-	.533**
	16.	TEAQ_AUT																-
HCs	1.	OTP_Other-directed	-	.822**	-.085	.004	.198	.113	-.034	-.078	-.050	.044	.054	.031	.086	.002	.222	.367*
	2.	OTP_Self-directed		-	-.064	.175	.081	.029	.117	-.001	.014	.114	.140	.196	.107	.075	.456**	.485**
	3.	MAIA_Noticing			-	-.261	-.526**	.263	.765**	.196	.351*	.036	.102	.245	.003	.095	.126	.039
	4.	MAIA_Not Distracting				-	.171	-.252	-.189	.034	.034	.407*	.249	.082	.077	.120	.264	-.032
	5.	MAIA_Not worrying					-	.057	-.543**	.060	-.351*	.050	-.078	-.150	.154	-.038	-.177	-.232
	6.	MAIA_Attention Regulation						-	.466**	.603**	.302	.462**	.071	.120	-.124	-.026	-.194	-.370
	7.	MAIA_Emotional Awareness							-	.453**	.585**	.215	.120	.224	-.078	.153	.191	-.120
	8.	MAIA_Self Regulation								-	.519**	.437**	-.060	.006	-.410*	-.108	-.217	-.523**
	9.	MAIA_Body Listening									-	.344*	.060	.090	-.442**	.104	-.012	-.254
	10.	MAIA_Trusting										-	.253	.258	-.212	.009	.075	-.121
	11.	TEAQ_FFT											-	.535**	.466**	.574**	.503**	.388*
	12.	TEAQ_CIT												-	.060	.523**	.572**	.320
	13.	TEAQ_ChT													-	.178	.262	.302
	14.	TEAQ_ASC														-	.398*	.353*
	15.	TEAQ_AIT															-	.571**
	16.	TEAQ_AUT																-

AN Current Anorexics, *RAN* Remitted Anorexics, *HCs* Healthy Controls, *OTP* Overall Touch Pleasantness, *MAIA* Multidimensional Assessment of Interoceptive Awareness, *TEAQ* Touch Experiences and Attitudes Questionnaire, *FFT* Family and Friends Touch, *CIT* Current Intimate Touch, *ChT* Childhood Touch, *ASC* Attitude to Self-Care, *AIT* Attitudes to Intimate Touch, *AUT* Attitudes to Unfamiliar Touch.

** p< .05*

*** p< .001*

Supplementary Information 2

COVID-19 touch experiences and eating behaviour Questionnaire

This unstandardized questionnaire consisted of 12 questions and was designed to measure touch experience and eating behaviour since the current COVID-19 pandemic. A 100-point VAS scale was used for participant responses and for each question. Participants could optionally provide additional comments concerning their responses. The questionnaire consisted of 6 questions concerning touch experiences, 1 for eating behaviour, 1 for physical activity rate, 1 question for social isolation, 1 question for the current country they live in, 1 question whether their country is currently in lockdown and 1 question for participants to state their level of risk of contracting COVID-19. Questions included: "To what extent has the level of touch you give to people within your household reduced since COVID-19?" and "Since COVID-19, I am feeling a great deal of social isolation from my usual support networks i.e., family and friends." This questionnaire was used to see whether changes in touch experiences since COVID-19, could account for any unexpected changes in touch pleasantness ratings (see below).

Results

Univariate Statistics

Table S2 demonstrates means and standard deviations for the Covid-19 subscales, which has been calculated separately for AN, RAN and HCs. The far-right column shows the output of pairwise comparisons between the three groups, which have been adjusted for multiple comparisons. HCs differed in their eating behaviours during the Covid-19 pandemic, reporting eating more than AN. Similarly, RAN also reported eating more during the Covid-19 pandemic than AN. During the pandemic, AN reported having done significantly more physical activity during the pandemic than RAN. All groups did not differ in any other Covid-19 subscale.

Table S2. Mean and standard deviation (in brackets) of demographics and Covid-19 subscale scores for AN (*n=27*), RAN (*n=29*) and HCs (*n=35*).

	AN (*n=27*) M (SD)	RAN (*n=29*) M (SD)	HCs (*n=35*) M (SD)	AN vs. RAN *p*	AN vs HCs *p*	RAN vs. HCs *p*
Given in Household (max 100)	15.56 (18.03)	24.89 (33.76)	15.88 (25.07)	*ns*	*ns*	*ns*
Received in Household (max 100)	17.04 (24.64)	23.85 (31.52)	17.85 (26.87)	*ns*	*ns*	*ns*
Given to Stranger (max 100)	67.38 (36.68)	66.86 (36.38)	82.38 (25.92)	*ns*	*ns*	*ns*
Received from Stranger (max 100)	64.67 (38.40)	64.45 (38.40)	76.46 (29.76)	*ns*	*ns*	*ns*
Level of touch reduced (max 100)	65.37 (28.02)	59.76 (32.17)	68.09 (24.54)	*ns*	*ns*	*ns*
Eating behaviours (max 100)	31.35 (20.34)	50.59 (25.17)	60.26 (18.29)	*.003*	*<.001*	*ns*
Physical activity rate (max 100)	54.00 (27.82)	35.69 (29.71)	44.77 (25.32)	*.021*	*ns*	*ns*
Social isolation (max 100)	58.31 (34.12)	53.07 (32.34)	55.41 (32.40)	*ns*	*ns*	*ns*

AN Current Anorexics*; RAN* Remitted Anorexics; *HCs* Healthy Controls; *M* Mean; *SD* Standard Deviation; *ns* Not Significant

Pleasantness of touch and the influences of the Covid-19 pandemic

For exploratory purposes, we then conducted Pearson's correlations with a Bonferroni correction amongst all self-report subscales to assess any association between Covid-19 with the PTA and OTP indices, separately for each group.

Pleasant Touch Awareness Correlations

We wanted, for exploratory purposes to investigate whether there was any association with touch avoidance and social isolation due to Covid-19 with pleasant touch awareness index (PTA) for each group, calculated separately per task.

For self-directed touch, Covid-19 subscales were not significantly associated with PTA for HCs (*all rs* > -.153, *ps* > .222). For AN, PTA was positively associated with a greater reduction in touch received in household (*r*= .537, *p*= .004) and more touch wanting during the Covid-19 pandemic (*r*= .388, *p*= .046). For RAN, PTA was negatively associated with levels of social isolation (*r*= -.481, *p*= .010).

For other-directed touch, Covid-19 subscales were not significantly associated with PTA for HCs (*all rs* > -.299, *ps* > .086). For AN, PTA was positively associated with greater Covid-19_eating behaviours (*r*= .441, *p*= .024). For RAN, Covid-19 subscales were not significantly associated with PTA (*all rs* > -.125, *ps* > .070).

To sum, pleasantness of CT-optimal touch involving the self and touch to another was largely influenced by Covid-19 for the AN group. Particularly, greater reduction in touch in household and touch wanting were associated with the pleasantness of touch for this group. Social isolation was a predictor of touch pleasantness for CT-optimal touch for RAN only. Covid-19 did not have an influence on touch pleasantness for CT-optimal touch for the HCs.

Overall Touch Pleasantness Correlations

We wanted, for exploratory purposes to investigate whether there was any association with touch avoidance and social isolation due to Covid-19 with overall touch pleasantness (OTP) for each group, calculated separately per task.

For self-directed touch, Covid-19 subscales were not significantly associated with OTP for HCs (*all rs* > -.009, *ps* > .291), AN (*all rs* > -.009, *ps* > .291) and RAN (*all rs* > -.229, *ps* > .056).

For other-directed touch, Covid-19 subscales were not significantly associated with OTP for HCs (*all rs* > -.282, *ps* > .107) and also for AN (*all rs* > -.192, *ps* > .079). For RAN, greater levels of touch wanting was positively associated with OTP (*r*= .376, *p*= .048).

To sum, Covid-19 did not have any negative influence on overall touch pleasantness for any of the groups. For both AN and HCs, Covid-19 did not influence overall pleasantness

judgements involving touch for another person. Only greater levels of touch wanting predicted overall touch pleasantness for RAN but no other group.

LIVERPOOL JOHN MOORES UNIVERSITY

COVID-19 Questionnaire

Title of Project: The observation of affective touch and the links with eating disorder behaviours.

COVID-19 Questionnaire

Please read and provide an answer to the following questions below. These questions concern your touch experiences, eating behaviours, physical activity rate and feelings of social isolation since the current COVID-19 situation. Please be as accurate as possible with your answers.

1. **What country do you currently live in?**

   ____

2. **Is the country you live in, currently in lockdown?**

   Yes (if yes, please state for how long)

   ____

   No

3. **Do you consider yourself at high risk of contracting COVID-19?**

   Yes
   No
   Unsure

4. **To what extent has the level of touch you give to people within your household reduced since COVID-19?**

   Not at all____Extremely

Comments (optional)

5. **To what extent has the level of touch you receive from people within your household reduced since COVID-19?**

Not at all____Extremely

Comments (optional)

6. **To what extent has the level of touch you give to a stranger reduced since COVID-19?**

Not at all____Extremely

Comments (optional)

7. **To what extent has the level of touch you receive from a stranger reduced since COVID-19?**

Not at all____Extremely

Comments (optional)

8. **Overall, since COVID-19, my level of touch with individuals has reduced.**

Not at all____Extremely

Comments (optional)

9. Currently, I want to be:

Touched by others less___________________________________Touched by others more

Comments (optional)

10. Since COVID-19, how would you best describe your eating behaviours?

Eating less____Eating more

Comments (optional)

11. Since COVID-19, my physical activity rates has:

Decreased____Increased

Comments (optional)

12. Since COVID-19, I am feeling a great deal of social isolation from my usual support networks i.e. family and friends.

Not at all____Extremely

Comments (optional)
